# Effect of composting on bacterial communities in poultry litter generated using conventional and organic production systems

**DOI:** 10.1002/jeq2.70240

**Published:** 2026-08-16

**Authors:** Danielli Monsores Bertholoto, Diogo Paes da Costa, Paula Fernanda Alves Ferreira, João Vitor da Silva Gonçalves, Ednaldo da Silva Araújo, Marco Antônio de Almeida Leal, Miliane Moreira Soares de Souza, Shana de Mattos de Oliveira Coelho, Irene da Silva Coelho

**Affiliations:** ^1^ Graduate Program in Science Technology and Innovation in Agriculture Federal Rural University of Rio de Janeiro Rio de Janeiro Brazil; ^2^ Microbiology and Enzimology Laboratory Federal University of Agreste Pernambuco Garanhuns Brazil; ^3^ Agronomy Soil Science Federal Rural University of Rio de Janeiro Rio de Janeiro Brazil; ^4^ Brazilian Agricultural Research Corporation Embrapa Agrobiology Rio de Janeiro Brazil; ^5^ Department of Microbiology and Veterinary Immunology, Institute of Veterinary Federal Rural University of Rio de Janeiro Rio de Janeiro Brazil

## Abstract

Poultry production generates substantial quantities of poultry litter, which can be applied to agricultural soils as organic fertilizer. Although this production system is sustainable, it can increase the microbial numbers and change ecological interactions and trophic networks in the soil. This study evaluated the composition, diversity, and structure of bacterial communities in poultry litter generated using conventional and organic production systems and subsequently composted under identical conditions for 125 days. Samples were collected before and after composting. Measurements included temperature, pH, electrical conductivity, and CO_2_ and NH_3_ emissions before and after composting. Total DNA was extracted, and the V3–V4 region of the 16S rRNA gene was sequenced using amplicon sequencing. The poultry litter reached temperatures around 65°C (±2°C) for 3 consecutive days, as established by Brazilian regulations for compost sanitization. The most abundant bacterial classes were Gammaproteobacteria and Alphaproteobacteria, representing 24.7% and 20.6% of the amplicon sequence variants, respectively. Among these groups, the orders Pseudomonales and Rhizobiales predominated. The order Enterobacterales was not detected in any of the poultry litter, before or after composting. Less abundant classes, such as Deltaproteobacteria, Bacilli, and Actinobacteria, showed reduced taxonomic diversity in both poultry litter after composting. Greater complexity and interconnection within the communities in both poultry litters were observed after composting. The bacterial community composition, diversity, and structure were influenced by both composting and the type of production system used. The impact of these remaining microorganisms on soil warrants further investigation because they can cause structural and functional changes in soil environments.

## Plain Language Summary

Chicken farms produce large amounts of litter that farmers use as organic fertilizer. However, this litter contains many bacteria, some potentially harmful. Composting is used to make chicken litter safer before applying it to soil. We investigated how composting affects bacterial communities differently in conventional farms (where antibiotics are used routinely) versus organic farms (where antibiotics are rarely used). Both types of litter reached 65°C during composting, meeting Brazilian regulations for the inactivation of pathogenic microorganisms. The production system influenced the initial bacterial composition, while composting had a stronger effect on shaping community structure. Composting safely reduces harmful bacteria in chicken litter for agricultural use. Understanding how different bacteria survive composting helps farmers use poultry waste more safely and sustainably.

**Abbreviations**
ASV: amplicon sequence variant
CPL: conventional poultry litter
EC: electrical conductivity
LDA: linear discriminant analysis
OPL: organic poultry litter
PCoA: principal coordinate analysis
SparCC: sparse correlations for compositional data

## 1 INTRODUCTION

Brazilian poultry production has achieved high levels of efficiency and has become a global reference in terms of production volume and export. Brazil is currently the second‐largest poultry producer worldwide and the leading exporter, accounting for approximately 4822 million tons per year (Associação Brasileira de Proteína Animal, 2023). However, poultry farms produce substantial amounts of poultry litter, particularly litter, which is used as bedding material. An agricultural census estimated that 1.25 billion chickens were produced in 2017, with each adult animal generating approximately 2 kg of poultry litter per year, resulting in a significant volume of waste material (Instituto Brasileiro de Geografia e Estatistica, 2017).

Using livestock manure as an organic fertilizer is considered the best option from agronomic, economic, environmental, and social perspectives (Corrêa et al., 2011). This practice aligns with Sustainable Development Goal 12, which aims to achieve sustainable waste management throughout the entire production cycle, adhering to international standards to minimize negative impacts on health and the environment (World Health Organization, 2015). In addition, Brazilian Federal Laws 10,831 of December 23, 2003, and 12,305 of August 2, 2010, recommend recycling organic waste to reduce the use of non‐renewable resources in agricultural systems (Brasil, 2003, 2010). Transforming livestock manure into organic fertilizer increases nutrient availability and organic matter content in the soil while decreasing the need for chemical fertilizers and lowering production costs (Verheijen et al., 1996; Weindorf et al., 2011).

Despite these benefits, poultry litter can also facilitate the development of microbial pathogens. The reuse of poultry litter in intensive production systems is common, leading to manure accumulation over time. The bacterial composition of poultry litter is similar to that of the natural microbiota of the chicken ileum, represented by the phyla Firmicutes, Proteobacteria, and Bacteroidetes (Kollarcikova et al., 2019). Several factors, including animal breed, age, gastrointestinal region, and husbandry practices, influence the intestinal microbiota of chickens. Among these practices, the use of antibiotics significantly impacts microbial composition.

In conventional animal production systems, antibiotics are used for disease treatment and prevention, as well as additives in subinhibitory concentrations to enhance metabolic efficiency and promote animal growth (Guardabassi & Kruse, 2010). Conversely, organic production systems operate under strict regulations regarding antibiotic use. The Brazilian Federal Government allows the use of antibiotics in organic production systems only when treatment with conventional drugs is ineffective and causes animal distress and death. The products, by‐products, and animal waste generated from animal production are considered organic materials after twice the grace period stipulated in the veterinary product leaflet, with a minimum of 96 h in all cases. Furthermore, antibiotics can be administered only once or twice in a 1‐year window, and animals requiring additional treatments are removed from the organic system (Ministério da Agricultura e Pecuária, 2011; Ministério da Agricultura e Pecuária, 2021). Consequently, these contrasting management approaches are expected to result in distinct microbial profiles in the poultry litter from each system.

Animal waste can be effectively used as an organic fertilizer; however, improper management can contribute to the development of pathogenic bacteria and negatively affect the structure and function of bacterial communities in different environmental reservoirs (Burki, 2018; Chen et al., 2017). Furthermore, increased bacterial concentrations of *Bacillus* spp. and *Pseudomonas* spp. in the soil, for example, can potentially influence soil ecological interactions, trophic networks, and biogeochemical cycles, which may lead to increased emissions of gases such as carbon dioxide, methane, and nitrous oxide and altering carbon and nitrogen cycles, depending on the specific species and environmental conditions  (Vieira, 2017). Effective treatment methods must be implemented to ensure the microbiological safety of poultry litter in agricultural systems. When conducted correctly, composting has several environmental benefits, such as reducing litter volume and odors, stabilizing organic matter, degrading toxic substances, and decreasing the number of potentially pathogenic microorganisms (Souza et al., 2019). Furthermore, composting is widely adopted by farmers to convert biomass waste into organic fertilizers due to its sustainability and low implementation and maintenance costs (Vicentin et al., 2021).

Although studies show that composting is an effective method for reducing the pathogen microbial load of chicken litter (Fioreze et al., 2020; Subirats et al., 2020), studies comparing the impact of management practices adopted in organic and conventional production systems on the microbiota of birds and, consequently, on the waste from these animals used as organic fertilizers in agricultural soils are still scarce. In fact, differences in feeding, in the use of antibiotics for therapeutic and prophylactic purposes, as well as in environmental conditions between organic and conventional production systems, can influence the microbial composition of poultry litter (Alexandrino et al., 2020; Cruz, 2019). This makes it essential to understand how these factors affect the safety of using organic fertilizers derived from these residues.

Our hypothesis is that differences in poultry management systems (conventional vs. organic) result in variations in the composition of the bacterial community in poultry litter, which are maintained and reflected in the microbial dynamics observed during the composting process. We believe that this study can contribute to a better understanding of the impacts of management on the microbiota of poultry litter and the composting process, providing support for safer and more effective practices in the reuse of these residues in agricultural systems. Given the above, the objective was to evaluate the composition, diversity, and structure of the bacterial community in poultry litter from the organic and conventional production systems, before and after the composting process.

## 2 MATERIALS AND METHODS

### 2.1 Poultry litter and composting

Poultry litter was collected from two farms using different production systems (conventional and organic) in the state of Rio de Janeiro, Brazil. The conventional poultry litter (CPL) was sourced from a farm located in Nova Friburgo (22°17′S and 42°32′W). This system involves the routine use of antibiotics (e.g., enrofloxacin, doxycycline, sulfaquinoxaline, and gentamicin for therapeutic purposes; enramycin, avilamycin, and virginiamycin as growth promoters) and houses approximately 10,000 broiler chickens per batch. The organic poultry litter (OPL) was obtained from an organic certified farm in São José do Vale do Rio Preto (22°09′S and 42°55′W). This farm adheres to organic standards, restricting antibiotic use to specific therapeutic cases as per Brazilian regulations, and houses approximately 2000 free‐range broiler chickens per batch (Ministério da Agricultura e Pecuária, 2011; Ministério da Agricultura e Pecuária, 2021). The poultry litter from both conventional and organic systems consisted primarily of wood shavings and other residual wood materials. The collected material represented the bulk litter discarded from each poultry house, without separation by flock cycle or bird age. In October 2019, approximately 4 m^3^ of litter from each production system was manually collected using shovels from multiple points distributed across the floor of the poultry houses to ensure a representative sample of the discarded material.

The collected material was transported by truck  to the Integrated Agroecological Production System (SIPA), where the composting experiments were conducted from October 2019 to March 2020. The SIPA is located in the municipality of Seropédica, a metropolitan region of the state of Rio de Janeiro, between coordinates 22°46′S and 43°41′W (Dias, 2007). The climate is hot and humid, classified as Aw, with high temperatures in the summer and mild temperatures in the winter. The mean annual temperature is 24.5°C, with concentrated rainfall from November to March and a mean annual precipitation of 1213 mm (Santos & Calderano, 1999).

Three replicate piles were constructed for each waste material (CPL and OPL), totaling six piles, in a covered warehouse. The piles were arranged in a cylindrical shape (height, 1.0 m; diameter, 1.2 m) using a galvanized wire mesh, which facilitated passive aeration through the lateral surface of the piles. Poultry litter was composted for 125 days. Piles were mechanically turned on days 14, 32, 60, 90, and 125 to promote active aeration. Moisture content was determined after each turning event, and the amount of water required to restore moisture to 45% was calculated based on the measured moisture content and the total mass of each pile; this amount was then added at the subsequent turning event. Samples were collected from each of three replicate piles (n = 3 per poultry litter type) before (day 0) and after (day 125) composting. Tree subsamples were collected at equidistant positions along each pile during turning, which were then thoroughly mixed to create composite samples. The samples were then divided for chemical analysis and DNA extraction.

### 2.2 Physicochemical analyses

Litter temperature was measured during the first 15 days of composting and subsequently every other day until the end of composting using a 22.5‐cm universal digital thermometer (model TP‐101; range, −50 to +300°C). Other analyses were conducted on days 0 and 125. pH was measured in distilled water solution at a 5:1 (v/v) ratio, and electrical conductivity (EC) was determined in the same aqueous extract, following Brasil (2007). Potential CO_2_ and NH_3_ emissions were used as indicators of compost stability and were quantified by incubating samples at 30°C for 72 h; gas capture was performed using NaOH and boric acid solutions for CO_2_ and NH_3_, respectively, following Leal (2020).

Data on the physicochemical parameters of poultry litter were compared between day 0 and day 125 within each poultry litter type. For each production system, conventional poultry litter and organic poultry litter were analyzed separately over time. The normality of the data was assessed using the Shapiro–Wilk test for each variable (pH, EC, CO_2_, and NH_3_), while the homogeneity of variances was verified using the Bartlett test. Then, the paired *t*‐test (p ≤ 0.05) was performed to compare the means between times.

The total concentrations of calcium (Ca), potassium (K), magnesium (Mg), and phosphorus (P) were determined to characterize the chemical composition of the poultry litter before and after the composting process (Table 1). Calcium and magnesium were quantified by atomic absorption spectrophotometry after acid digestion, potassium by flame photometry, and phosphorus by a colorimetric method using molybdovanadate (Nogueira & Souza, 2005).

**TABLE 1:** Total (T) and soluble (S) levels of calcium (Ca), potassium (K), magnesium (Mg), and phosphorus (P) on poultry litter generated using conventional and organic production systems on days 0 and 125 of composting.

	Ca T (g kg^−1^)	K T (g kg^−1^)	Mg T (g kg^−1^)	P T (g kg^−1^)	Ca S (cmolc dm^−^^3^)	K S (mg L^−1^)	Mg S (cmolc dm^−^^3^)	P S (mg L^−1^)
**Conventional poultry litter**								
Day 0	14.72 ± 1.69	16.64 ± 0.57	3.39 ± 0.07	7.13 ± 0.25	2.16 ± 0.12	1596.56 ± 150.36	2.10 ± 0.12	499.59 ± 201.68
Day 125	38.02 ± 16.14	27.75 ± 2.58	5.00 ± 0.71	11.17 ± 1.05	2.17 ± 0.22	2802.80 ± 235.29	2.56 ± 0.17	876.14 ± 142.57
**Organic poultry litter**								
Day 0	19.15 ± 1.83	16.63 ± 0.51	3.28 ± 0.22	8.08 ± 1.01	1.91 ± 0.03	1697.33 ± 53.37	2.30 ± 0.40	492.94 ± 58.25
Day 125	32.83 ± 14.33	25.95 ± 1.52	4.90 ± 0.76	12.06 ± 0.64	1.76 ± 0.28	2698.83 ± 150.92	2.01 ± 0.04	983.39 ± 93.83

*Note*: Values are the means ± standard deviation of triplicates.

### 2.3 Total DNA extraction, library preparation, and 16S rRNA gene sequencing

Genomic DNA from the litter samples was extracted using the Power Soil DNA Isolation Kit (Qiagen Inc.) following the manufacturer's instructions. DNA samples were sent to Macrogen (Seoul, South Korea) (www.dna.macrogen.com) for library preparation and sequencing. The V3‐V4 region of the 16S rRNA gene was sequenced using the universal primers Bakt341F (5′‐CCTACGGGNGGCWGCAG‐3′) and Bakt805R (5′‐GACTACHVGGGTATCTAATCC‐3′), as described previously (Herlemann et al., 2011). Sequencing was performed using Herculase II Fusion DNA Polymerase (Agilent Technologies Inc.) and the Nextera XT v2 Index Kit (Illumina Inc.) following the manufacturer's guidelines. Sequencing was performed on an Illumina MiSeq sequencing platform using the v3 flow cell. A library concentration of 3 pM was loaded, with a 30% increase in the concentration of control DNA (Illumina PhiX), following the manufacturer's recommendations. Raw data were converted to the FASTQ format using bcl2fastq version 2.20 (Illumina). The sequences were demultiplexed, and the barcodes were removed.

### 2.4 Processing of raw data

Raw data consisted of 1,315,312 sequence pairs (forward and reverse) across 11 amplicon libraries in FASTQ format. Adapters were removed, and sequences were trimmed and filtered based on quality scores ≥30 using the Deficiency of Adenosine Deaminase 2 pipeline version 1.16 in R version 3.6.3 and RStudio version 1.4.1717 (Callahan et al., 2016; RStudio Team, 2022). The FIGARO tool was used for optimizing trimming parameters (Weinstein et al., 2019). Forward and reverse sequences were truncated at 263 and 222 nucleotides, respectively, and sequences were discarded if the maxEE was greater than 4 or 3. Sequences were also truncated when the quality score (truncQ) was ≤2. The read error rates were estimated using the “learnErrors” function, alternating error rate estimation and sample inference until convergence. Amplicon sequence variants (ASVs) were inferred using the “dada” function, and the samples were grouped using the standard method. Forward and reverse sequences were merged using the “mergePairs” function. Chimeras were removed using the “removeBimeraDenovo” function and the consensus method. Taxonomic classification was assigned to the phylum and genus levels based on the Silva SSU v132 database using the IDTAXA algorithm in the DECIPHER package, which performs better than the naive Bayesian method (Murali et al., 2018; Quast et al., 2013; Wright, 2016). The sequences of chloroplasts, mitochondrial genomes, and any other non‐bacterial origin were removed. After data processing, 398,559 sequences corresponding to 8192 ASVs from bacterial taxa were obtained. The ASV data were combined into a single object using the “phyloseq package” in R, allowing the selection of up to 30,400 reads per sample, and a rarefaction step was performed to normalize sequencing depth.

### 2.5 Statistical analysis

Bacterial community structures were evaluated via permutational analysis of variance and distance‐based redundancy analysis of covariance matrices and Bray–Curtis distances, based on ASV counts and environmental data, using the vegan package version 2.5.7 in R (Oksanen, 2020). The abundance of unique, shared, and exclusive ASVs among the three niches was measured using the multinomial species classification method, based on a specialization threshold of the greatest majority (*K* = 2/3). To account for multiple comparisons, the significance level for individual tests was set to *p *= 0.001, as recommended by Chazdon et al. (2011) for assemblage‐wide classifications to control for experiment‐wise error.

The abundance of ASVs in each niche was evaluated using linear discriminant analysis (LDA). Statistical analysis was performed using the non‐parametric Kruskal–Wallis test, followed by Dunn's post hoc test. *p*‐values of less than 0.05 were considered statistically significant. For network analysis, ASVs were first identified at the phylum level. ASVs that appeared at least four times were included in the analysis. The most significant interactions between these filtered ASVs in CPL and OPL, and between ASVs before and after composting, were evaluated using bootstrap estimates of SparCC correlation coefficients in the SpiecEasi package version 1.1.0 in R (Kurtz et al., 2015). Bootstrap‐based SparCC correlations were estimated in R using the sparccboot function from the SpiecEasi package, generating the node and edge matrices used for network construction (Kurtz et al., 2015). Edges were retained when they showed significant correlations (*p* < 0.01) and absolute correlation weights ≥0.7. The filtered network matrices were subsequently imported into Gephi software version 0.9.2, where the sign of the SparCC correlation coefficient was used to classify associations as positive or negative, and node‐level topological parameters, including the number of connections (degree) and betweenness centrality, were calculated using the software's built‐in network analysis tools. Network graphs were also constructed in Gephi (Bastian et al., 2009). The remaining multivariate analyses and calculations of indices and correlations were performed using the vegan package (Oksanen, 2020). Graphs not related to network visualization were constructed using the ggplot2 package in R version 3.3.3 (Wickham, 2009).

## 3 RESULTS

### 3.1 Physicochemical characteristics of poultry litter during composting

**FIGURE 1:**
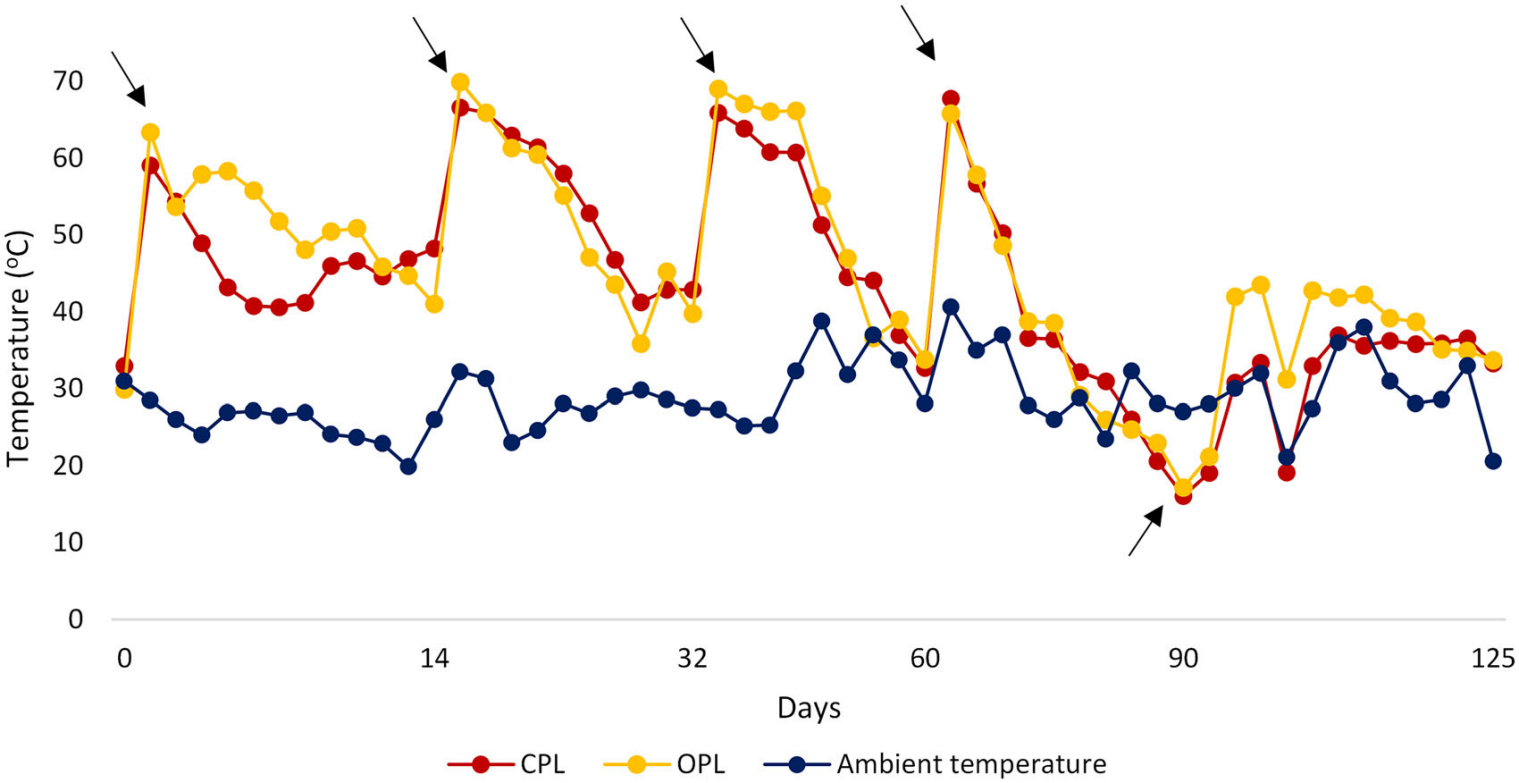
Temperature variations during poultry litter composting using conventional and organic production systems. The arrows indicate the maximum and minimum temperatures in each sample. CPL, conventional poultry litter; OPL, organic poultry litter.

The composting process of the residues was conducted for 125 days. During this period, the temperature fluctuation patterns were similar for both composts, reaching peaks of up to 65 and 67°C for CPL and OPL, respectively. Temperature peaks were observed at 3, 15, 33, and 61 days. The minimum values were recorded at day 90, when temperatures in conventional and organic poultry litter reached 16 and 17°C, respectively (Figure 1). The temperature peaks observed at 3, 15, 33, and 61 days were associated with the irrigation and aeration of the composts performed on the preceding days (0, 14, 32, and 60 days).

The pH of poultry litter increased significantly between day 0 and day 125 in both systems evaluated. EC increased significantly in OPL, whereas in CPL, it did not differ between the evaluated time points.

Potential CO_2_ emissions decreased significantly between day 0 and day 125 in both poultry litter types. NH_3_ emissions were also significantly reduced in CPL and OPL after composting (Table 2). No relevant changes in particle size or physical structure were observed throughout the composting process, as both CPL and OPL presented a fine texture from the beginning of composting.

**TABLE 2:** pH values, electrical conductivity (EC), and potential CO_2_ and NH_3_ emissions on days 0 and 125 of composting poultry litter generated using conventional and organic production systems.

	pH	EC (µS cm^−1^)	CO_2_ (mg g^−1^)	NH_3_ (mg^−^^1^ NH_3_ g DM day^−^^1^)
**Conventional poultry litter**				
Day 0	8.7 ± 0.03	2.54 ± 0.28	20.67^a^ ± 1.78	1.29^a^ ± 0.20
Day 125	9.7^a^ ± 0.04	2.38 ± 0.17	7.71 ± 0.57	0.02 ± 0.01
**Organic poultry litter**				
Day 0	9.30 ± 0.12	1.99 ± 0.21	22.53^a^ ± 1.39	0.85^a^ ± 0.13
Day 125	10.0^a^ ± 0.10	2.42^a^ ± 0.08	7.46 ± 0.67	0.00 ± 0.00

*Note*: Values are the means of triplicates plus the standard deviation.

^a^ Indicates a significant difference between day 0 and day 125 within the same poultry litter type, according to the paired *t*‐test at 5% significance. Abbreviation: DM, dry matter.

### 3.2 Changes in bacterial community structure and composition

**FIGURE 2:**
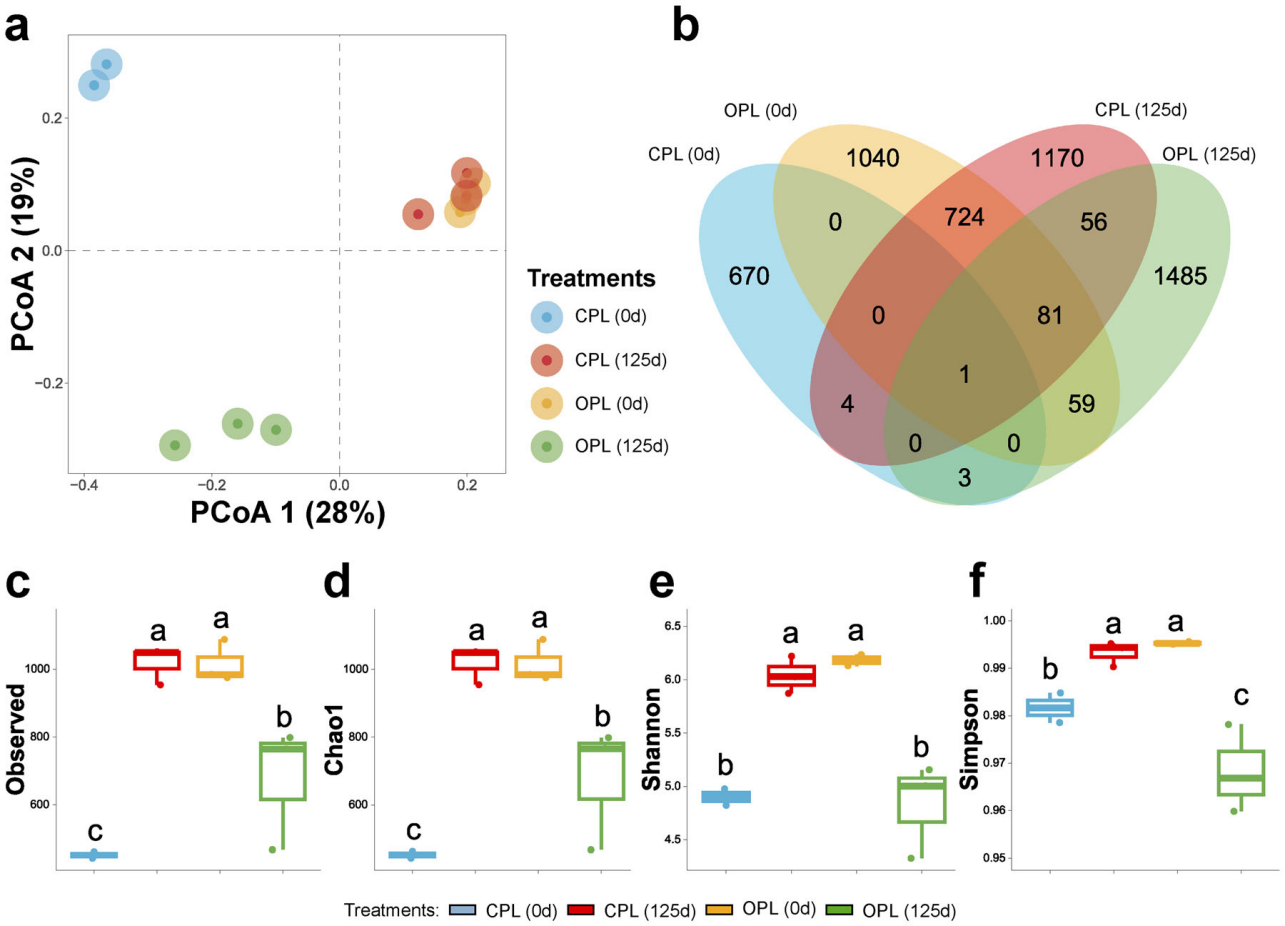
Bacterial community structure and alpha diversity in composted and uncomposted poultry litter generated using conventional and organic production systems. (a) Principal coordinate analysis (PCoA) based on UniFrac distances (beta‐diversity). (b) Venn diagram showing the amplicon sequence variants (ASVs) among groups. (c) Observed number of ASVs (richness); (d) Chao‐1 richness estimator; (e) Shannon–Wiener diversity index; and (f) Simpson's diversity index. In the boxplots, groups not sharing the same lowercase letter differ significantly according to Tukey's honestly significant difference (HSD) test, with *p*‐values adjusted using the Benjamini–Hochberg method (*p* < 0.05). Each boxplot is based on three biological replicates (*n* = 3 per group), corresponding to independent composting piles. CPL, conventional poultry litter; OPL, organic poultry litter.

The results of the principal coordinate analysis (PCoA) revealed that the microbial community structure was more strongly affected by the composting process than by the type of production system (Figure 2a). The most distinct communities were observed between CPL on day 0 and OPL on day 125, with the multivariate model explaining 47% of the total variance. Community structures were similar between composted CPL and uncomposted OPL, indicating that the bacterial taxa naturally present in uncomposted OPL were also present in composted CPL.

The numbers of shared and exclusive ASVs are shown in Figure 2b. During composting, the number of exclusive ASVs increased by 75% (from 670 to 1170) in CPL and by 43% (from 1040 to 1485) in OPL. One ASV was common to all samples and treatments, while 806 ASVs were shared between composted CPL and uncomposted OPL, of which 724 were unique to this comparison. No ASVs were shared between CPL and OPL before composting, whereas 128 ASVs were shared between CPL and OPL after composting, 56 of which were unique to this comparison. This indicates that composting favors the development of specific bacterial taxa, independent of the source material.

Analysis of alpha diversity revealed that the observed number of ASVs was the highest in composted CPL and uncomposted OPL, intermediate in composted OPL, and the lowest in uncomposted CPL (Figure 2c). The same pattern was observed for Chao‐1 richness (Figure 2d). The Shannon–Wiener index was similar between composted CPL and uncomposted OPL, and both were significantly higher than uncomposted CPL and composted OPL (Figure 2e). For the Simpson index, composted CPL and uncomposted OPL also showed the highest diversity values, whereas uncomposted CPL showed intermediate values and composted OPL showed the lowest values (Figure 2f). Therefore, composting increased bacterial richness and diversity in CPL but reduced these indices in OPL.

**FIGURE 3:**
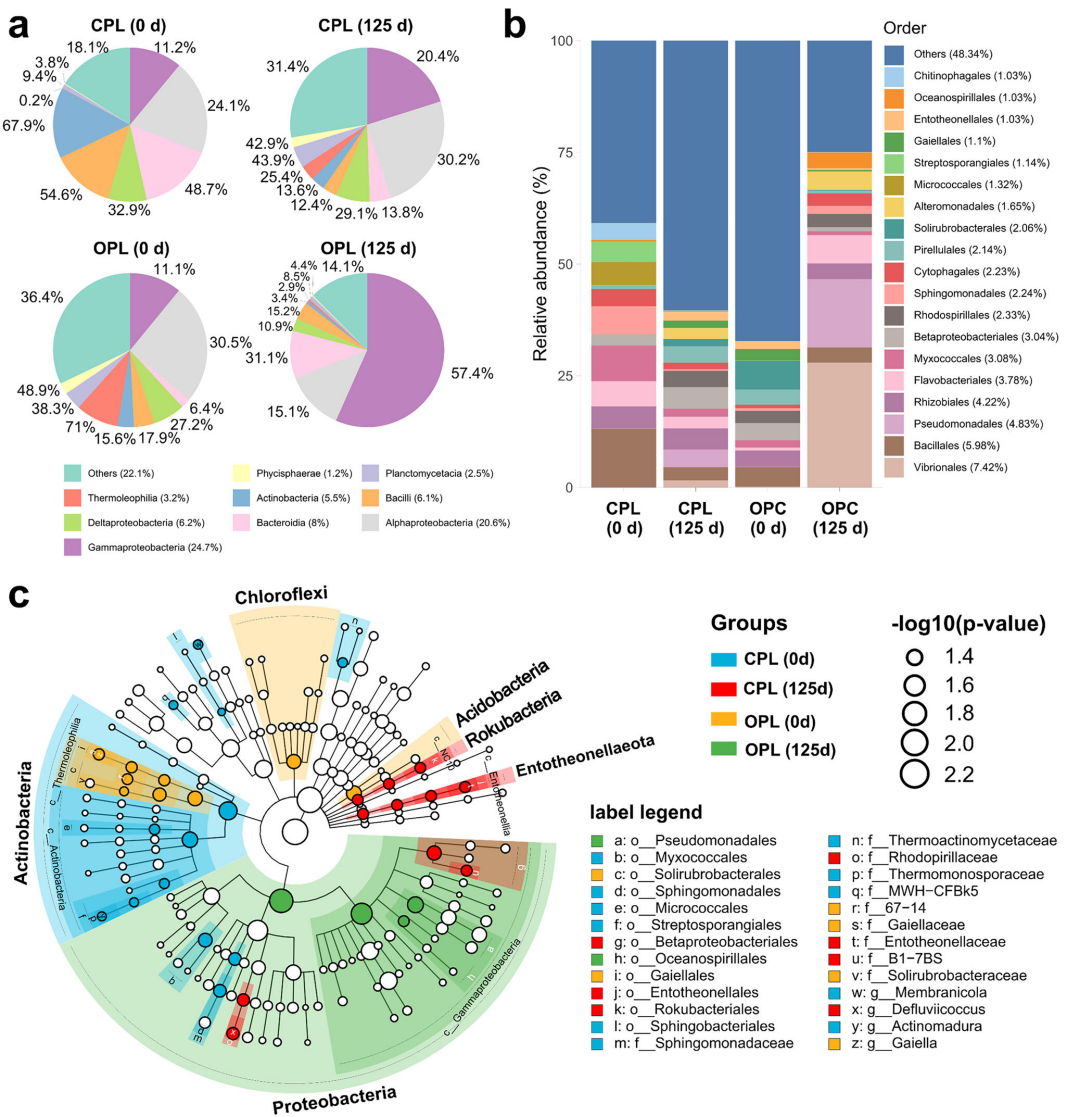
Taxonomic composition of bacteria in composted and uncomposted poultry litter. (a) Ten most abundant classes. (b) Twenty most representative orders (abundance greater than 1%). (c) Linear discriminant analysis (LDA) of significantly enriched taxa across samples. In LDA, differences in taxonomic distribution and midpoints were assessed using the non‐parametric Kruskal–Wallis and Wilcox post hoc tests, respectively. *p*‐values were adjusted using the Benjamini–Hochberg method (*p* < 0.05). Colors indicate the representativeness of each sample's taxa (circles) and phylogenetic branches.

The relative abundance of the nine most representative classes, 19 most representative orders (relative abundance higher than 1%), and remaining taxa (designated “others”) are shown in Figure 3. Gammaproteobacteria and Alphaproteobacteria were the most abundant classes, representing 24.7% and 20.6% of ASVs, respectively. Composting increased the relative abundance of Gammaproteobacteria, particularly in OPL. The relative abundances of the classes Deltaproteobacteria, Bacilli, Actinobacteria, Thermoleophilia, Planctomycetacia, Phycispaerae, and others were 6.2%, 6.1%, 5.5%, 3.2%, 2.5%, 1.2%, and 22.1%, respectively (Figure 3a).

In the class Gammaproteobacteria, the relative abundances of Vibrionales, Pseudomonales, Alteromonadales, and Oceanospirillales were 7.42%, 4.83%, 1.65%, and 1.03%, respectively. In Alphaproteobacteria, the relative abundances of Rhizobiales and Rhodospirillales were 4.22% and 2.33%, respectively (Figure 3b). Linear discriminant analysis demonstrated that the phyla Actinobacteria and Gammaproteobacteria were enriched in the uncomposted and composted OPLs, respectively (Figure 3c).

### 3.3 Analysis of co‐occurrence

**FIGURE 4:**
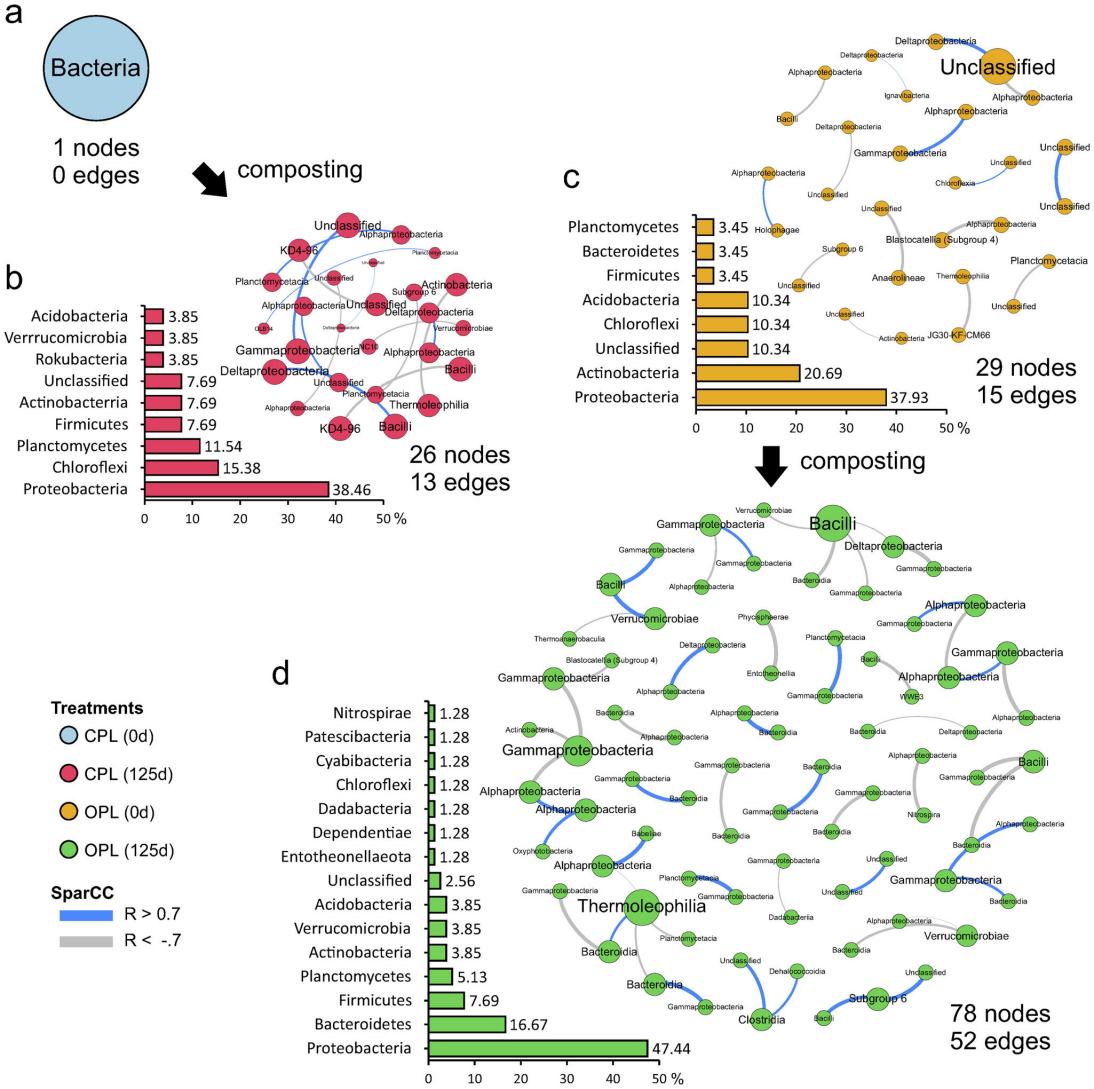
Sparse correlations for the spatial correlations for compositional data (SparCC) analysis of the effects of composting on the bacterial network in conventional and organic production systems. (a) Node of a bacterial network in the conventional system, showing the absence of significant interactions (two‐sided pseudo *p* < 0.05 based on bootstrapping with100 repetitions). (b) Bacterial network in the conventional system after composting. (c, d) Bacterial network in the organic system before and after composting. Nodes represent the amplicon sequence variants (ASVs) involved in significant co‐abundance and spatial correlations for compositional (SparCC) modules greater than 0.7, labeled at the taxonomic class level. Node size is proportional to the number of connections (weighted degree by SparCC), and edge thickness is proportional to the SparCC value.

Co‐occurrence analysis showed that composting strongly affected the number of taxa and their interconnections in microbial networks (Figure 4, Table 3). Composting increased the complexity of significant interactions (*p* < 0.01), with positive and negative correlations having weights above 0.7, except in the uncomposted CPL (Figure 4a), where no connections or taxa predominated, based on the filtering criteria used in spatial correlations for compositional data (SparCC). However, composting increased the representativeness (percentage of nodes) of certain taxa in CPL (Figure 4b), particularly Proteobacteria (38.5%), Chloroflexi (15.4%), Planctomycetes (11.5%), Firmicutes (7.7%), and Actinobacteria (7.7%), which formed a network containing 26 nodes (ASVs) and 13 interactions (edges = SparCC).

**TABLE 3:** Network metrics of bacterial communities in poultry beds from organic and conventional systems before and after the composting process.

	CPL day 0^a^	CPL day 125	OPL day 0	OPL day 125
Number of edges	–	26	29	78
Number of vertices	–	13	15	52
Positive vertex	–	54%	40%	52%
Average degree^b^	–	1	1.034	1.333
Average path degree^c^	–	0.902	0.938	1.292
Network diameter^d^	–	1	2	5
Density^e^	–	0.02	0.037	0.017
Connected components^f^	–	13	14	26
Modularity^g^	–	0.923	0.923	0.936
Average path length^h^	–	1	1.062	1.867

Abbreviations: CPL, conventional poultry litter; OPL, organic poultry litter.

^a^ CPL day 0, uncomposted conventional poultry litter; CPL day 125, conventional poultry litter after 125 days of composting; OPL day 0, uncomposted organic poultry litter; OPL day 125, organic poultry litter after 125 days of composting.

^b^ Number of direct connections with other nodes.

^c^ Number of direct connections to other nodes, where the degree of the nodes is weighted by edge correlation.

^d^ The longer of the pairwise shortest path lengths or the size of the largest connected component.

^e^ Measure of the integrity and effectiveness of the network, representing the observed fraction of actual connections relative to possible collections.

^f^ Number of nodes with connections.

^g^ Modularity is a measure of a network or graph structure that quantifies the strength of the division of a network into modules. Networks with high modularity exhibit dense connections among nodes within modules and sparse connections between nodes in different modules, with a higher value indicating a stronger division of the network into modules.

^h^ Clustering coefficient and short average geodesic distance, indicators of system performance or the degree of compaction of the microbial structure. That is, this measures the average number of steps required to move from one node to another in the network.

In OPL, composting increased the network from 29 nodes and 15 interactions to 78 nodes and 52 interactions (Figure 4c,d). The most representative phyla in uncomposted OPL were Proteobacteria (39.9%), Actinobacteria (20.7%), Chloroflexi (10.3%), and Acidobacteria (10.3%). The predominance of the phyla Proteobacteria, Bacteroidetes, Firmicutes, Planctomycetes, and Actinobacteria in uncomposted OPL was 47.4%, 16.7%, 7.7%, 5.1%, and 3.8%, respectively; these compositions and structures were similar to those in composted CPL (Figure 4b). Composting increased the number of significant positive interactions from 40% to 52% in OPL and increased the network diameter, number of interconnections, and average path length, indicating higher complexity (Table 3).

## 4 DISCUSSION

During the composting process, CPL and OPL showed a very similar temperature pattern (Figure 1). The maximum temperature peaks were observed at 3, 15, 33, and 61 days of composting. These peaks were associated with humidification and pile turning performed on the preceding days, corresponding to days 0, 14, 32, and 60, which promoted aeration and consequently supported microbial activity and greater heat release.

After each temperature peak, the subsequent cooling phase was likely associated with a decrease in oxygen diffusion caused by the natural settling and compaction of the piles, together with a possible reduction in moisture availability. These conditions may have limited microbial activity and, consequently, reduced heat production. As a result, the composting process was characterized by alternating thermophilic and mesophilic phases. This temperature behavior is consistent with the pattern described by Oviedo‐Ocaña et al. (2015). The temperatures reached by the poultry litter were high during composting. Conselho Nacional do Meio Ambiente (CONAMA) resolution No. 481/2017 establishes that to reduce the microbial load of organic solid waste during composting in open systems, a temperature of 55°C must be maintained for 14 consecutive days, or a temperature of 65°C must be maintained for 3 consecutive days (CONAMA, 2017). Thermophilic temperatures during composting indicate high microbial metabolic activity and the mineralization of organic nitrogen, as evidenced by increased NH_3_ concentrations and elevated pH levels. This suggests that the alkaline pH in both types of litter at the beginning and end of composting is associated with organic matter degradation and the concentrations of produced, converted, or volatilized ammonia (Ezzariai et al., 2018; Leal et al., 2011). In the initial stages, microorganisms that can tolerate higher temperatures and an alkaline environment predominate, such as thermophilic bacteria, which are responsible for accelerated decomposition. As the process progresses, conditions become more favorable for microorganisms that are better adapted to lower temperatures and less extreme environments, such as mesophilic bacteria and fungi, which results in a change in the composition and diversity of the microbiome (J. Ma et al., 2025).

Organic matter stabilization is typically associated with a decline in pile temperature to near‐ambient levels and a reduction in microbial activity, often reflected by decreased CO_2_ evolution and NH_3_ dissipation (Wichuk & McCartney, 2010). At 125 days of composting, the temperature of the litter in both production systems decreased, and CO_2_ and NH_3_ emissions were also reduced, suggesting the end of the stabilization period and the beginning of the curing or maturation period. In the last stage of composting, the poultry litter reaches optimum physical, chemical, and biological characteristics and is thus ready to be used as fertilizer in agricultural soils (Kiehl, 2012; Leal, 2020).

PCoA and diversity analysis showed differences in the bacterial community composition of uncomposted poultry waste between the two production systems (Figure 2). Although chicken microbiota has unique communities throughout the gastrointestinal tract, the composition of less abundant bacterial taxa varies depending on the type of husbandry practice (Cheng et al., 2025; Muyyarikkandy et al., 2023). Antibiotics used to prevent and treat diseases, increase feed digestibility, and improve animal health and performance can alter the microbial composition of both chicken microbiota and litter (Alexandrino et al., 2020). In this study, the routine use of antibiotics for therapeutic and growth‐promoting purposes in the conventional system may have inhibited the development of specific bacterial taxa, decreasing species richness in uncomposted CPL compared to that in uncomposted OPL.

Composting altered the bacterial community structure and composition, increasing the diversity in CPL and decreasing the diversity in OPL. During composting, bacterial activity decomposes organic matter, increasing the sample temperature to approximately 65°C (Ezzariai et al., 2018). Temperature is one of the main factors driving changes in the microbial community during composting. The persistence of certain microorganisms in composted poultry litter is also relevant. Although composting effectively reduces pathogenic bacteria, some resistant taxa, such as Clostridium and Enterococcus, were detected in the final poultry litter compost, suggesting that specific conditions may favor their survival (Gurtler et al., 2018). This emphasizes the importance of optimizing composting parameters, including aeration and moisture control, to improve pathogen suppression and ensure the microbiological safety of the final product.

The predominant phyla in chicken intestinal microbiota are Firmicutes, Bacteroidetes, Proteobacteria, and Actinobacteria (Oakley et al., 2014; Shang et al., 2018; Xiao et al., 2017; Zhao et al., 2019). Consistent with the literature, all four phyla were detected in both CPL and OPL before and after composting. The phylum Proteobacteria was the most abundant in both production systems before and after composting, and the classes Gammaproteobacteria and Alphaproteobacteria were the most representative, corresponding to 24.7% and 20.6% of the total number of ASVs, respectively. The predominance of Proteobacteria in poultry litter is determined by the rearing conditions that affect animal health, well‐being, and age, and this taxon is more prevalent in young chickens (Kollarcikova et al., 2019; Ocejo et al., 2019). However, since the collected material represented bulk discarded litter without separation by flock cycle or bird age, a direct association between bird age and Proteobacteria abundance cannot be established in the present study.

Across the complete dataset, the class Gammaproteobacteria represented 24.7% of the ASVs, with the order *Pseudomonales* accounting for 4.83% of the total ASVs. However, this percentage was not constant across all samples, since the relative abundance of Pseudomonales varied among treatments. Pseudomonales were found in composted CPL and OPL. In contrast, this order was absent in uncomposted CPL and low in uncomposted OPL. Pathogenic strains of *Pseudomonas* in poultry litter pose potential health risks to humans and animals and can negatively impact the environment by causing infectious diseases, producing toxins, and promoting antibiotic resistance (Ferreira, 2021). However, not all *Pseudomonas* species are pathogenic, and several species degrade organic matter, thereby improving soil nutrient cycling and fertility. Furthermore, certain species can help control plant pathogens by competing for nutrients and space and by producing antibiotics (Awasthi et al., 2018; S. S. Ma et al., 2018; Sandini et al., 2019; Wang et al., 2015).

Many human pathogens in the bacterial order Enterobacterales cause food safety risks in composted manures, but this order was not found in any of the poultry litter samples, before or after composting (Puño‐Sarmiento et al., 2014). Enterobacterales were not detected among the ASVs identified in the CPL and OPL samples, either before or after composting. This result suggests that this bacterial order was not abundant or was below the detection threshold of the sequencing approach used in this study. However, because this study did not quantify viable pathogens or assess food safety indicators, these results should not be interpreted as evidence of pathogen elimination. Thus, the non‐detection of Enterobacterales suggests a lower potential risk associated with this bacterial order, but it does not confirm microbiological safety of the final compost.

Among Alphaproteobacteria, the second most representative class in our samples, the order Rhizobiales was the most abundant across the complete dataset, accounting for 4.22% of the total ASVs. However, this percentage should not be interpreted as constant across all samples, since the relative abundance of Rhizobiales varied among treatments. This order was found in both CPL and OPL before and after composting. Species in this order fix atmospheric nitrogen, converting it to a form usable by plants. This increases soil fertility and nitrogen availability for plant growth and reduces the need for nitrogen fertilizers (Babalola, 2010). Among the less abundant classes Deltaproteobacteria (6.2%), Bacilli (6.1%), and Actinobacteria (5.5%), the taxonomic diversity of orders containing potentially pathogenic taxa, such as Myxococcales, Bacillales, and Micrococcales (Figure 3c), decreased in both production systems after composting.

Co‐occurrence analysis showed that, after composting, taxa from the phyla Proteobacteria, Planctomycetes, Firmicutes, and Actinobacteria were predominant in both systems, suggesting that composting more efficiently recruited species belonging to the four main phyla in both systems and other less frequent phyla, including Verrucomicrobia and Acidobacteria. After composting, the communities exhibited higher complexity and interconnection, with OPL showing more ecological interactions than CPL (52 vs. 13), indicating a more complex pattern of potential associations among bacterial taxa. Although previous studies have linked microbial network complexity with organic matter decomposition and community organization (Banerjee et al., 2016; Martins, 2021), our network analysis does not directly measure decomposition rates, resilience, or pathogen suppression.

Poultry litter generated in conventional and organic production systems exhibited distinct bacterial community compositions. We believe that these differences influenced the outcome of composting, which altered the bacterial composition but did not eliminate all microbial groups, despite its role as a sanitation method. Nonetheless, larger studies are needed to assess the impact of microorganisms in organic fertilizers on agricultural soils, as they can cause structural and functional changes that affect soil ecology and nutrient cycling.

## AUTHOR CONTRIBUTIONS

**Danielli Monsores Bertholoto**: Conceptualization; formal analysis; methodology; writing—original draft; writing—review and editing. **Diogo Paes da Costa**: Formal analysis; methodology; writing—original draft; writing—review and editing. **Paula Fernanda Alves Ferreira**: Formal analysis; methodology; writing—original draft; writing—review and editing. **João Vitor da Silva Gonçalves**: Formal analysis; methodology; writing—original draft. **Ednaldo da Silva Araújo**: Conceptualization; writing—original draft; writing—review and editing. **Marco Antônio de Almeida Leal**: Conceptualization; writing—original draft; writing—review and editing. **Miliane Moreira Soares de Souza**: Conceptualization; funding acquisition; project administration; supervision; writing—review and editing. **Shana de Mattos de Oliveira Coelho**: Conceptualization; funding acquisition; writing—review and editing. **Irene da Silva Coelho**: Conceptualization; funding acquisition; investigation; supervision; writing—review and editing.

## CONFLICT OF INTEREST STATEMENT

The authors declare no conflicts of interest.

## Data Availability

The genetic sequences and corresponding Bio Sample assignments from this study can be accessed in the NCBI repository, under project code PRJNA 1167323.
